# Utility of melatonin on brain injury, synaptic transmission, and energy metabolism in rats with sepsis

**DOI:** 10.1007/s00210-024-03337-8

**Published:** 2024-08-06

**Authors:** Gulten Ates, Sule Tamer, Elif Ozkok, Hatice Yorulmaz, Gul Ipek Gundogan, Abdullah Aksu, Nuray Balkis

**Affiliations:** 1 Department of Physiology, Faculty of Medicine, Istanbul Yeni Yuzyil University, Yilanlı Ayazma St, Cevizlibag, Istanbul, 34010 Turkey; 2 Department of Physiology, Istanbul Medical Faculty, Istanbul University, Istanbul, Turkey; 3 Department of Neuroscience, Aziz Sancar Institute of Experimental Medicine, Istanbul University, Istanbul, Turkey; 4 Faculty of Health Science, Halic University, Istanbul, Turkey; 5 Department of Histology and Embryology, Faculty of Medicine, Biruni University, Istanbul, Turkey; 6 Department of Chemical Oceanography, Institute of Marine Sciences and Management, Istanbul University, Istanbul, Turkey

**Keywords:** Sepsis, Melatonin, Brain damage, Synaptophysin, Neuron-specific enolase, Energy

## Abstract

Melatonin is a powerful endogenous antioxidant hormone. Its healing effects on energy balance and neuronal damage associated with oxidative metabolism disorders have been reported in pathologic conditions. We aimed to determinate the utility of melatonin on neuronal damage, synaptic transmission, and energy balance in the brain tissue of rats with sepsis induced with LPS. Rats was divided into four groups such as control, LPS (20 mg/kg i.p.), melatonin (10 mg/kg i.p. × 3), and LPS + Melatonin (LPS + Mel). After 6 h from the first injection, rats were decapitated, and also tissue and serum samples were taken. Lipid peroxidation and neuron-specific enolase (NSE) levels were determined from the serum in all group. High energy compounds, creatine, and creatine phosphate are measured by HPLC methods from the homogenized tissue. Counts of living neurons are marked with NeuN (neuronal nuclei), degenerated neurons are marked with S100-ß and synaptic vesicles transmission is analyzed with synaptophysin antibodies immunoreactivities. One-way ANOVA and post hoc Tukey tests were used to statistical analysis. In LPS group, AMP, ATP, creatine, and creatine phosphate levels were significantly decreased (*p* < 0.05), and also ADP levels were significantly increased compared with the other groups (*p* < 0.01). Living neurons counts were significantly decreased in LPS (*p* < 0.01), melatonin, and LPS + Melatonin (*p* < 0.05) groups compared with control. Degenerated neurons counts were increased in LPS group compared with control (*p* < 0.01) and also decreased in both of melatonin and LPS + Melatonin groups (*p* < 0.01). Synaptophysin immunoreactivity was decreased in LPS group compared with the other groups (*p* < 0.05). We observed that melatonin administration prevents neuronal damage, regulates energy metabolism, and protects synaptic vesicle proteins from sepsis-induced reduction.

## Introduction

Sepsis is a life-threatening organ dysfunction syndrome characterized by dysregulated immune responses to infection (Singer et al. 2016). Its worldwide incidence has been reported to be more than 30 million cases each year (Fleischmann et al. 2016). Recent studies have suggested that pro-inflammatory mediators and sepsis-induced oxidative stress directly cause dysfunction in enzyme complexes in the mitochondrial respiratory chain, resulting in various pathophysiological disorders on energy balance and metabolism (Vanasco et al. 2012; Karapetsa et al. 2013; Ates et al. 2022). Increased superoxide production with mitochondrial dysfunction in sepsis has been shown to contribute to the remission phase of oxidative damage in various ischemic organs or tissues, especially in the early time after sepsis diagnosis, causing high oxidative stress and low antioxidant potential activity (Hsiao et al. 2020).

Mitochondria have been reported to play a main role in sepsis-associated redox dysregulation. A reversible bioenergetic disturbance has been observed in sepsis, resulting in impaired oxygen consumption and hyperlactatemia due to mitochondrial dysfunction (Singer 2014). The kidney, heart, and brain are the organs with the highest mitochondria density and are most susceptible to sepsis-induced mitochondrial dysfunction (Veltri et al. 1990). Organ dysfunctions are an important complication of sepsis, and sepsis-induced brain dysfunctions are a very common and observable finding in the early stages. Brain dysfunction is caused by a diversity of factors that frequently occur during sepsis. Brain dysfunction caused by sepsis in the early phase manifests itself with sepsis-associated encephalopathy (SAE), delirium, cerebral ischemia, and bleeding (Pan et al. 2022; Hassan et al. 2022).

Production of oxygen radicals such as reactive oxygen species (ROS), nitric oxide (NO), and peroxynitrite by neutrophils and macrophages induce gene expression of pro-inflammatory mediators. Increased ROS production may be responsible for tissue damage in septic shock and endotoxemia. It has been shown in studies that melatonin and its metabolites can clear ROS/RNS (reactive nitrogen species). Melatonin has additional advantages than other antioxidants in preventing oxidative damage (Tan et al. 2015). Since melatonin reaches high concentrations in mitochondria and together with its metabolites, it has a powerful antioxidant effect that protects mitochondria from oxidant damage, and it is known the strongest endogenous antioxidant molecule (Colunga Biancatelli et al. 2020).

It has been reported that mitochondrial dysfunction, which develops as a result of inflammatory and oxidative damage due to sepsis, induces disruptions in the production of high-energy components and their derivatives such as adenosine triphosphate (ATP), which causes disorders in cellular energy metabolism, resulting in neuronal damage and tissue death (Karapetsa et al. 2013; Xu et al. 2019; Ates et al. 2022) The level of developing neuronal damage can be analyzed with acute biomarkers that can be examined in serum, such as neuron-specific enolase (NSE) (Yao et al. 2014; Bangshøj et al. 2022). In experimental studies, the degree of neuronal damage and its effect on neuronal transmission can be revealed by marking live and degenerate neurons in samples taken and also by detecting proteins that have a role in neuronal transmission, such as synaptophysin (Granja et al. 2021).

LPS is a cell wall component of gram-negative bacteria. It triggers the pathways that initiate the inflammatory response and oxidative stress and also could lead to multiple organ dysfunction and mortality (Tan et al. 2015; Ates et al. 2022).

In our study, we aimed to examine the effects of melatonin on brain damage, energy metabolism, and synaptic transmission in rats with sepsis induced with LPS.

## Materials and methods

### Experimental groups and procedures

Ethical approval for the research was received from Istanbul Bağcılar Training and Research Hospital Animal Experiments Local Ethics Committee (2016/62). The animals were fed trade diet and tap water ad libitum and were housed in cages maintained at controlled temperature (22 ± 2 °C) and humidity (55–60%) and a 12-h light/dark cycle. Adult male *Sprague Dawley* rats (180–230 g) were separated into four groups as controls (*n* = 8), LPS (*n* = 8) (20 mg/kg, i.p.), melatonin (*n* = 8) (10 mg/kg, i.p. × 3), and LPS + Melatonin (LPS + Mel) (*n* = 8) (Nezić et al. 2009).

**Fig. 1:**
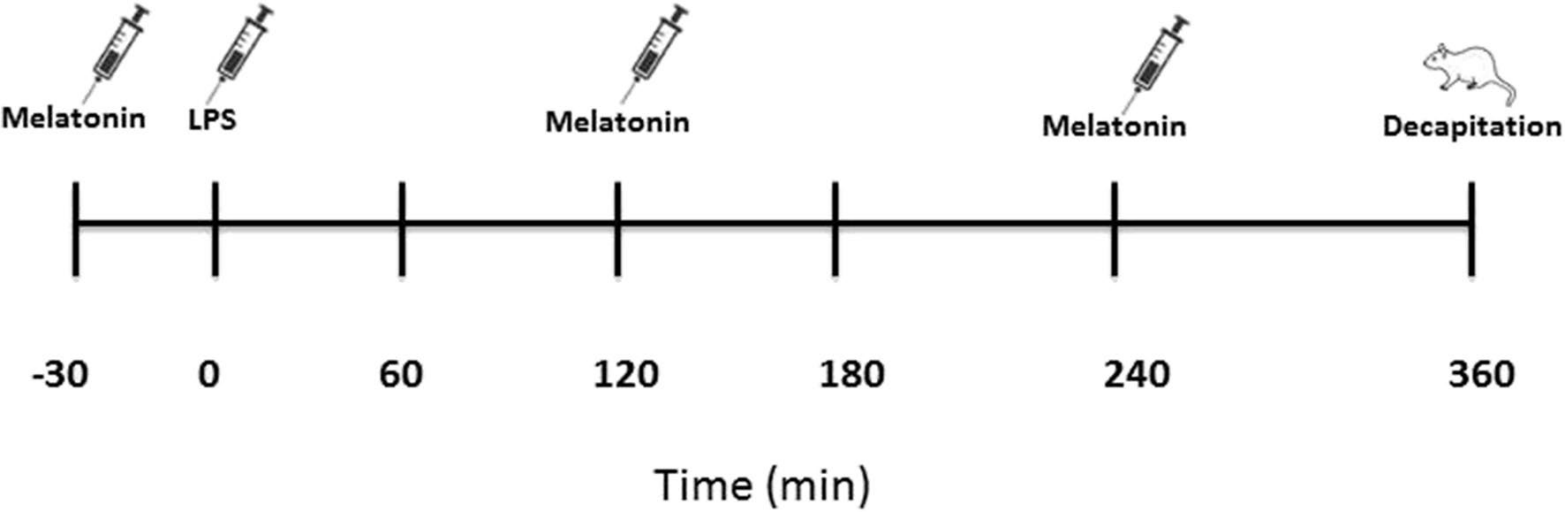
Diagram of experimental procedure

Melatonin applied 10 mg/kg i.p. doses for three times at 2-h intervals (Sigma Aldrich, Product No: M5250). In the LPS + Melatonin group, a dose of melatonin was injected 30 min before the LPS administration. After LPS application, doses of melatonin were applied twice, at 2nd and 4th hours (Fig. 1) (Wu et al. 2001).

LPS from *Escherichia coli* O55: B5 (Sigma Aldrich, Product No: L2880) was dissolved in 1 mL of sterile saline solution, and a single i.p. dose of 20 mg/kg was injected. After 6 h from the LPS application, all experimental groups were sacrificed under the anesthesia (xylazine + ketamine by i.p. routes), and blood samples and brain tissues were taken from all rats. The brain tissue samples were rapidly frozen by liquid nitrogen for analysis of creatine, creatine phosphate, and high energy compounds. The remaining parts of the same tissue samples were separated in 10% formaldehyde for histological and immunofluorescence examinations.

### Determination of high energy compounds, creatine, and creatine phosphate levels

#### Sample preparation

Brain tissue samples weighing 250 mg were homogenized in 2 ml 0.42 M HClO4 using a homogenizer (Ultraturrax T25) for 30 s. A 1.0 ml supernatant was taken for adjusting pH with 1.0 M K2HPO4 after centrifugation at 3000 rpm for 5 min.

#### Analyzing by HPLC

Adenine nucleotides (adenosine monophosphate (AMP), adenosine diphosphate (ADP), ATP), creatine and creatine phosphate were analyzed with C18 column (5 μm, 250 mm × 4.6 mm, Nucleodur, USA) with isocratic elution using a KOH/KH2PO4 buffer (215 mM, pH 6.25), 3 mM tetrabutylammonium phosphate, and 5% acetonitrile ion-paired reverse-phase chromatography using HPLC (Agilent 1100, USA) at 214 nm (Sellevold et al. 1986). Creatine phosphate, creatine, and adenine nucleotides were calculated from their external standard curves from different concentration phases.

### Determination of lipid peroxidation

Lipid peroxide levels in the serum samples were estimated by measuring thiobarbituric acid reactive substance (TBARS) levels. Serum samples were reacted with thiobarbituric acid (TBA, Sigma Aldrich) reagent containing trichloroacetic acid (TCA, Sigma), concentrated hydrochloric acid (HCl, Merck) and butylated hydroxytoluene (Sigma), boiled for 15 min, then cooled, and was centrifuged. TBARS levels were analyzed in the supernatant fraction at 532 nm using spectrophotometry (Shimadzu, Japan), and concentrations were calculated and exhibited as nmol/ml.

### Determination of neuron-specific enolase levels

Approximately 5 cc of blood from the heart of anesthetized rats was taken into a dry tube for neuron-specific enolase (NSE) analysis. The blood collected in dry tubes was centrifuged for 10 min at 3000 rpm, and the serum phase was separated into Eppendorf tubes and taken to Istanbul Medical Faculty Department of Biochemistry along with the blood in EDTA tubes for evaluation. Serum samples were studied with autoanalyzers (Roche Cobas Integra Systems, Roche Diagnostic/Germany).

### Immunofluorescence protocol

Before staining, the hippocampus region was marked from 10-µm serial sections and preparations for staining. Overnight, brain tissue was preserved in 10% formaldehyde. In subsequent steps, tissue was treated for 24 h in a succession of solutions with lowering alcohol concentrations to eliminate any residual fixative. After removing the alcohol, the tissues were treated with xylene for 1 h to guarantee that the paraffin wax was well absorbed. For 2 h, tissues were stored in pure paraffin at 58 °C. The tissues were embedded in paraffin when they were prepared for blocking. Using a microtome, 10-μm-thick pieces of the blocks were removed and placed on slides coated with poly-l-lysine for staining.

Neuronal degeneration was detected with using the S100-ß antibody (orb388636) biorbyt, Unipoert ID: P04271, Entrez: 6285, Mouse, (secondary antibody Dylight 649, goat anti-mouse IgG (H&L) Abbkine #A23610). NeuN (neuronal nuclei) antibody (orb48522) biorbyte, Unipoert ID: A6NFN3, Entrez: 146713, Rabbit (second-ary antibody green-FITC) was utilized to identify live neurons. To detect synaptophysin, SYP (orb69251) biorbyte, Entrez: 6855, Mouse (secondary antibody Dylight 649, goat anti-mouse IgG (H&L) Abbkine #A23610) was used.

The sections underwent multiple fluorescence staining procedures. Following the paraffin purification process, xylene was vaporized until the sections turned white. The parts were then left to stand in − 20 °C cold methanol for 10 min. Lastly, the portions were submerged in distilled water for 2 min to rehydrate. Epitopes were then boiled in a microwave oven for 20 min using 1 × citrate buffer (citrate buffer, pH 6.0) after incubating with 0.2% Tween 20 for 5 min and cleaning with deionized water. Sections were rinsed twice with phosphate-buffered saline (PBS; pH 7.6) for 10 min at room temperature. NeuN was diluted with PBS containing 2% serum and sprinkled onto the sections before the primary antibodies were tuned for dilution. The sections were then covered with a coverslip and stored at + 4 °C for the all night. After 2-h incubation with the room temperature, secondary antibody of the NeuN was removed and placed onto sections containing either 10% goat serum diluted in PBS or 0.3% TritonX-100 diluted in PBS containing 0.3% serum. The sections were then covered and stored overnight at + 4 °C. After 2-h incubation with the room temperature next day, the secondary antibodies of the primary antibodies were cleaned with PBS. Dapi was used to incubate the core dye, and anti-fading and mounting media were used to seal the preparations. FITC, H&L, and DAPI-compatible filters were used in fluorescence microscopy to assess and investigate histologic changes.

### Statistics

The findings obtained in the study will be analyzed statistically using IBM Statistical Package for the Social Sciences (SPSS) 22.0 (SPSS, Inc., Chicago, IL, USA). All parametric data were evaluated by one-way analysis of variance (ANOVA) and Tukey test, and non-parametric data were evaluated by Kruskal–Wallis analysis of variance and the Mann–Whitney *U* test. The post hoc Tukey test was used to determine the group that caused the difference. Data are given out as the mean ± standard error of the mean (SEM). *p* < 0.05 was considered as statistically significant.

## Results

### Findings of high energy compounds and creatine and creatine phosphate

There were significantly decrease in levels of ATP of total brain tissues compared with the control, melatonin, and LPS + Mel groups (*p* < 0.001) and also levels of creatine p compared with control, melatonin, and LPS + Mel (both, *p* < 0.05) in LPS group. In LPS + Mel group, ATP levels decrease compared with the control group (*p* < 0.05).

**Fig. 2:**
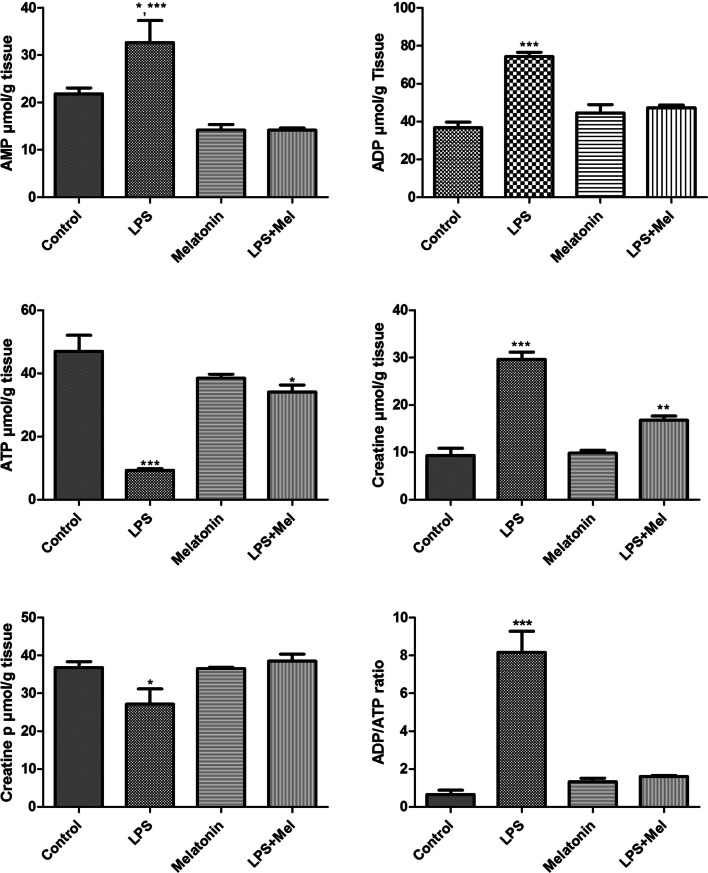
AMP, ADP, ATP, creatine, creatine p levels, and ADP/ATP ratio in the total brain tissue were measured with HPLC method in all experimental groups: control, LPS (20 mg/kg i.p.), melatonin (10 mg/kg × 3 doses i.p.), and LPS + Mel (LPS group treated with melatonin). **a** *p < 0.05 LPS vs. control group; ***p < 0.001 LPS vs. melatonin and LPS + Mel groups. **b** ***p < 0.001 LPS vs. control, melatonin, and LPS + Mel groups. **c** *p < 0.05 LPS + Mel vs. control group; ***p < 0.001 LPS vs. control, melatonin, and LPS + Mel groups. **d** *p < 0.01 LPS + Mel vs. control and melatonin groups; ***p < 0.001 LPS vs. control, melatonin, and LPS + Mel groups. **e** *p < 0.05 LPS vs. control, melatonin, and LPS + Mel groups. **f** ***p < 0.001 LPS vs. control, melatonin, and LPS + Mel groups

On the other hand, there were significantly increasing in AMP, ADP, creatine levels, and ADP/ATP ratio in LPS group compared with the control (for AMP levels *p* < 0.05 vs. LPS group), melatonin, and LPS + Mel (for creatine levels *p* < 0.01 vs. control and melatonin groups) groups (both, *p* < 0.001) (Fig. 2a–f).

### Findings of lipid peroxidation

**Fig. 3:**
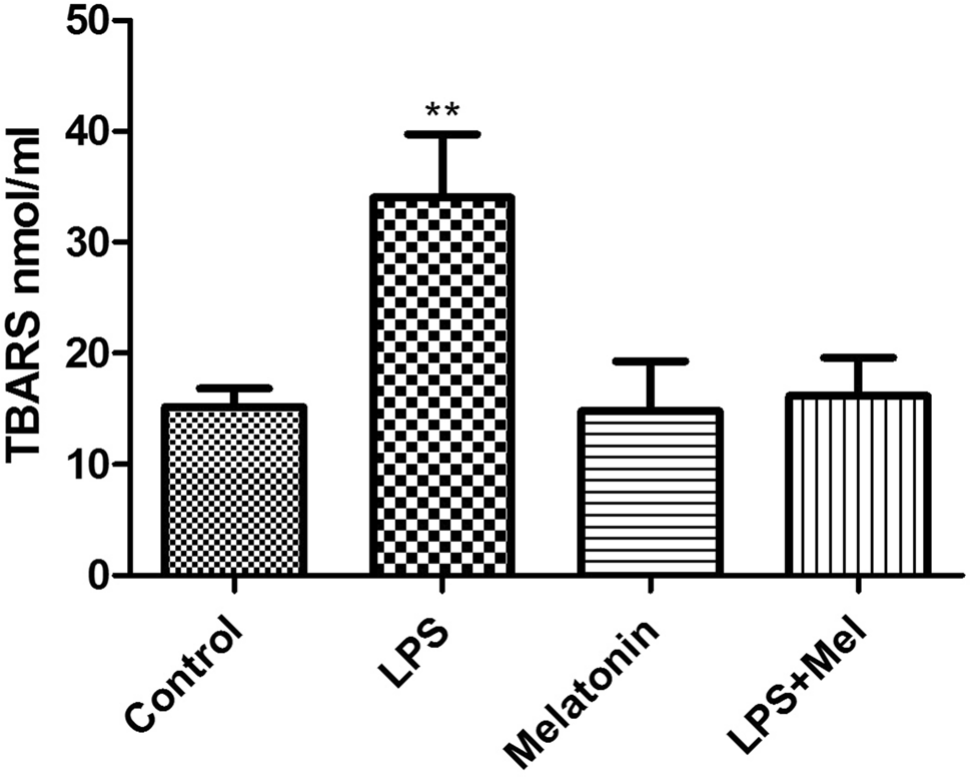
Lipid peroxidation (TBARS) levels (nmol/ml) of all experimental groups: control, LPS (20 mg/kg i.p.), melatonin (10 mg/kg × 3 doses i.p.), and melatonin + LPS (LPS group treated with melatonin). **p < 0.01 LPS vs. control, melatonin, and LPS + Mel groups

There were significantly increase in the serum lipid peroxidation levels in the LPS group, compared with the other experimental groups (*p* < 0.01); also, there is no significantly changing between control, melatonin, and LPS + Mel groups (*p* > 0.05) (Fig. 3).

### Findings of neuron-specific enolase (NSE)

**Fig. 4:**
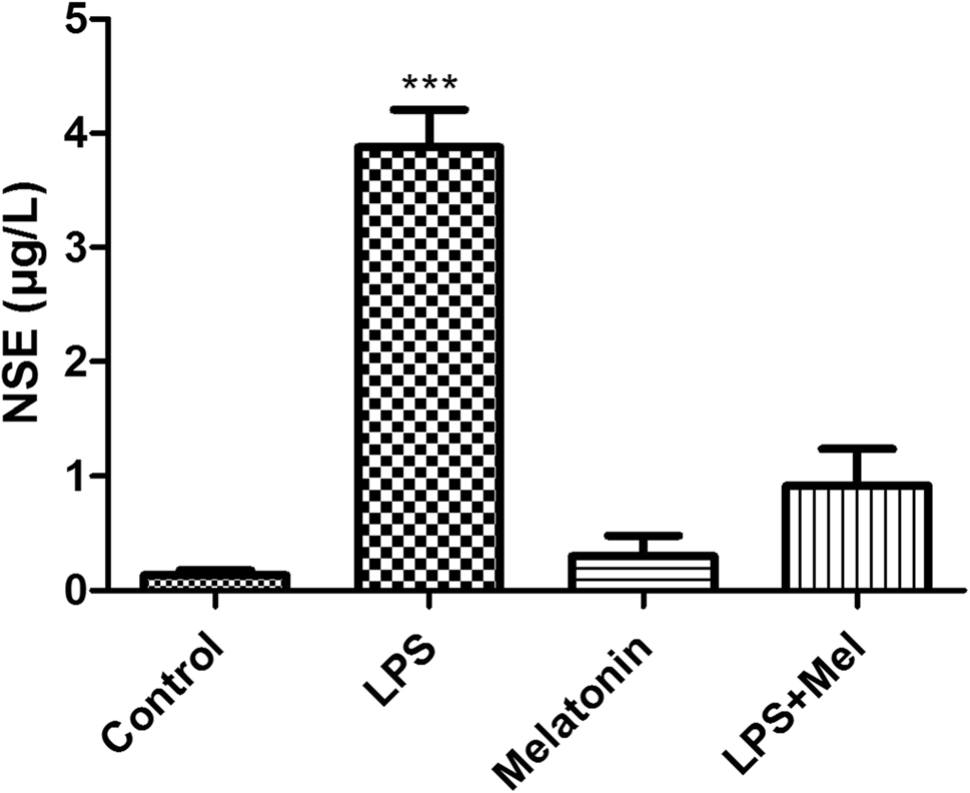
Serum NSE levels (μg/L) in all experimental groups. ***p < 0.001 LPS vs. control, melatonin, and LPS + Mel groups

In LPS group, there were significantly increasing in the serum NSE levels compared with the other experimental groups (*p* < 0.01). No significantly change was detected between the control, melatonin, and LPS + Mel groups (*p* > 0.05) (Fig. 4).

### Findings of immunofluorescence staining

There were significantly differences in levels of the S100-ß immunoreactivity which were marked degenerated neurons. In LPS group, neuron counts marked with S100-ß significantly increased compared with the other experimental groups in hippocampus region of brain sections (*p* < 0.001).

**Fig. 5:**
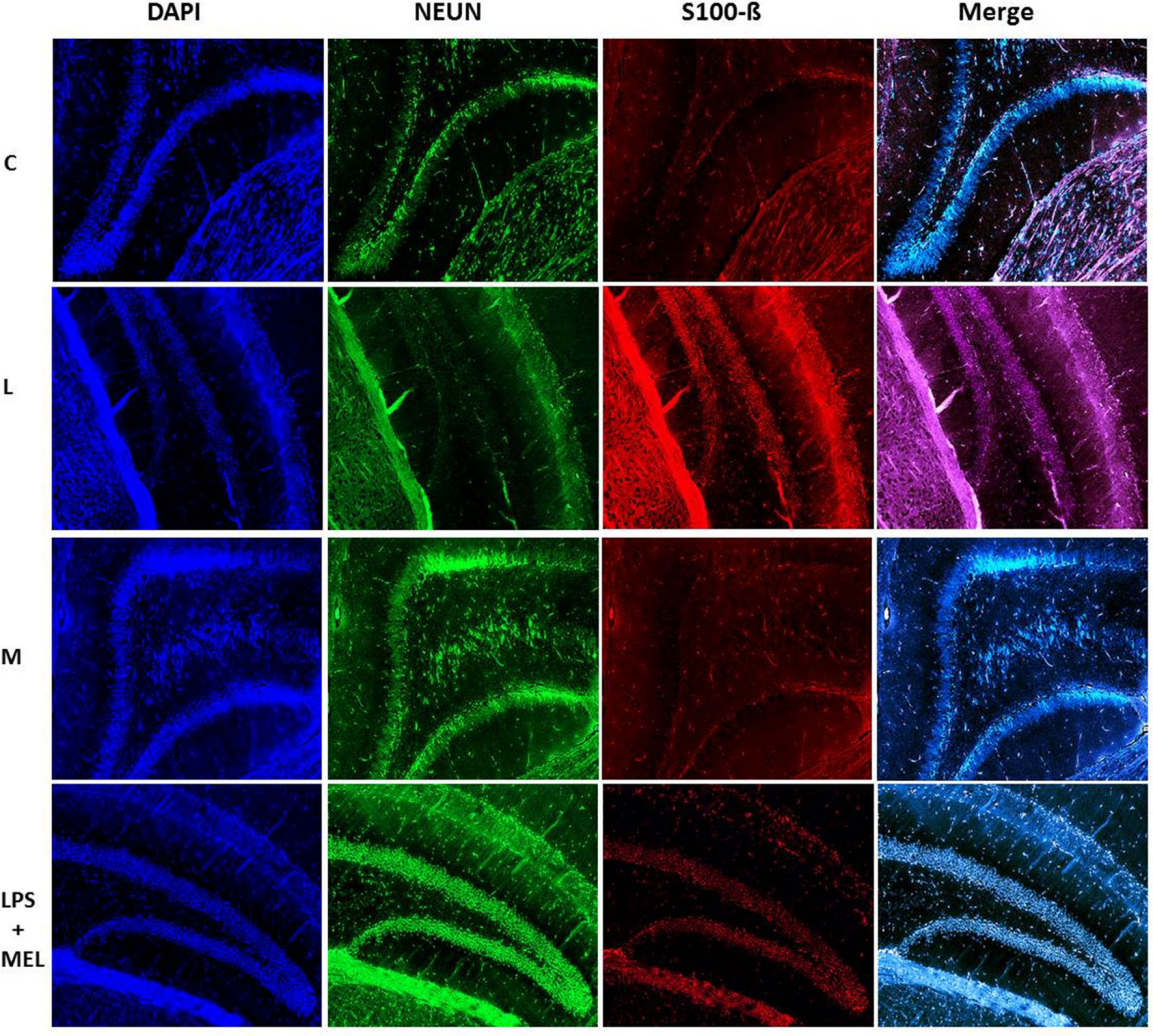
Hippocampus region of the brain tissue sections were stained in experimental groups with DAPI, NEUN, and synaptophysin antibodies × 10; (C) control, (L) LPS, (M) melatonin, and LPS + Mel group, 100 μm scale bar

**Fig.6:**
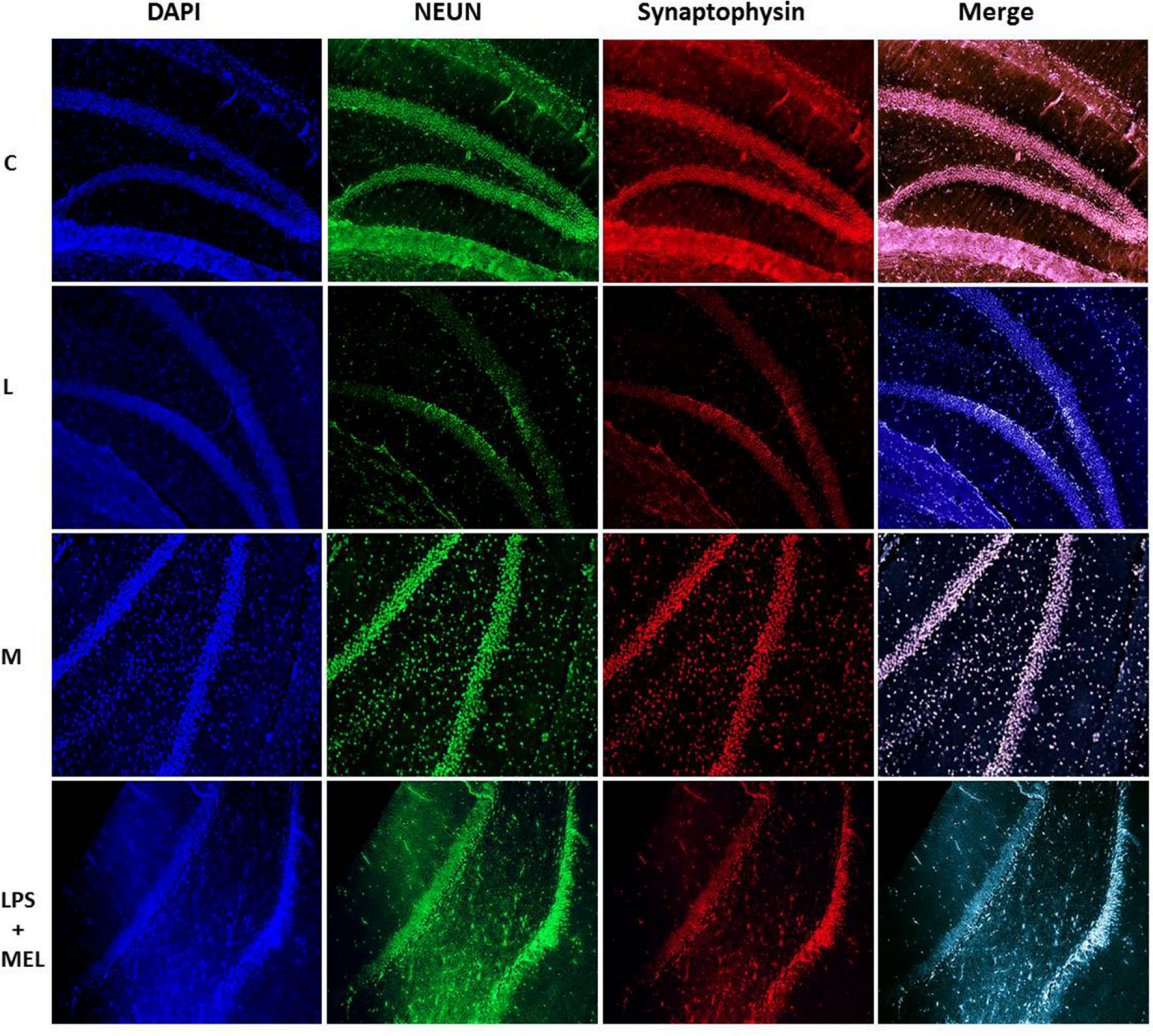
Hippocampus region of the brain tissue sections were stained in experimental groups with synaptophysin antibody × 10 and × 40 magnifications, (C) control, (L) LPS, (M) melatonin, and LPS + Mel group, 100 μm scale bar

**Fig. 7:**
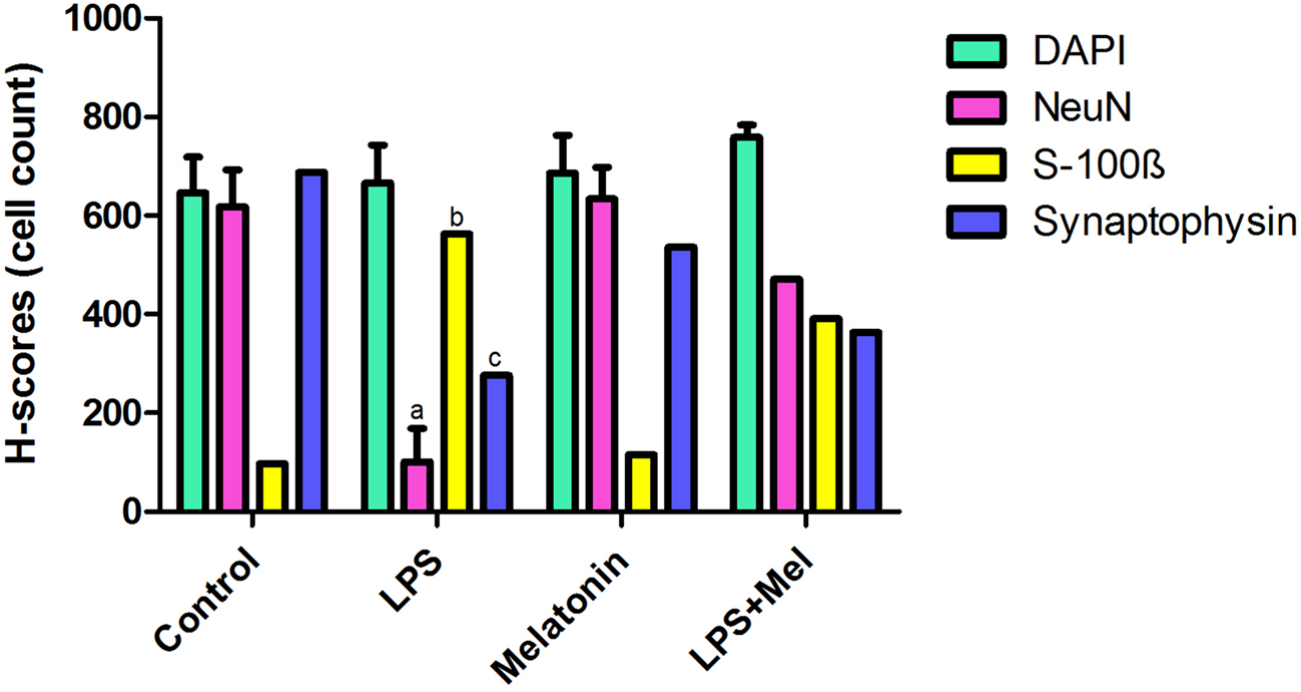
H-scores of sections of the hippocampus region of the brain tissue’s immunoreactivity from the all group. **a** *p* < 0.01 LPS vs. all experimental groups for NeuN; **b** *p* < 0.01 LPS vs. all experimental groups for S100-ß; **c** *p* < 0.01 LPS vs. all experimental groups for synaptophysin

There were significantly differences in levels of the NeuN which were marked living neurons and synaptophysin involvement in hippocampus region of brain sections. In LPS group, neuron counts marked with antibodies of NeuN and synaptophysin were found decreased compared with the other experimental groups (*p* < 0.01). No significant changes were determined among other groups (*p* > 0.05) (Figs. 5, 6 and 7).

## Discussion

Sepsis is characterized by oxidative stress that occurs when the balance between oxidants and antioxidants is disturbed. In patients with severe sepsis, antioxidant capacity may be compromised, and increased levels of metabolic products of free radical damage can be observed (Zhang et al. 2022). Oxidative stress is one of the essential factors contributing to cerebral biochemical impairment. The central nervous system is more sensitive to oxidative stress. This may be due to many factors, such as neurons using greater amounts of oxygen, iron concentrations being higher in many parts of the brain, neuronal membranes having polyunsaturated fatty acids that are sensitive to oxidative stress, and neuronal mitochondria producing large amounts of hydrogen peroxide (Hassan et al. 2022).

Numerous articles in the literature have shown that pro-inflammatory cytokines and oxidative stress cause sepsis tissue damage and mortality. Many studies reported that pro-inflammatory cytokines enter the brain during sepsis by several mechanisms. LPS can activate TLRs through areas and damage blood–brain barrier (BBB), such as the hippocampus, choroid plexus, and circumventricular organ. Also in the literature notice that many cytokines enter the brain through receptor-mediated endocytosis on brain endothelial cells that is activated. Activation of cytokine receptors such as IL-1 receptor (IL-1R1) and TNF receptor (TNFR) elevates cytokine levels in the brain. Once these inflammatory mediators enter the brain tissue, microglial cells get activated. Once microglia are activated, they most likely induce brain damage during sepsis. Sustained microglia activation enhances the production of inflammatory cytokines and reactive oxygen species (ROS), which creates a vicious cycle of increased BBB permeability and increased neuronal apoptosis (Sekino et al. 2022). It has been reported that melatonin reduces tissue necrosis by reducing malondialdehyde and myeloperoxidase expression levels in the liver, brain, lung, and kidneys (Zhang et al. 2017). Recent studies have been suggested that melatonin’s attenuating effect on brain lipid peroxidation production was mediated by nitric oxide neutralization (Al-Olayan et al. 2015). Additionally, another study reported that melatonin decreased lipid peroxidation by increasing superoxide dismutase and catalase levels in the brain hippocampus tissue (Kotler et al. 1998). In parallel with literature, in our study, we determined the increased levels of TBARS in LPS group compared with the other experimental group. On the other hand, in LPS + Melatonin group, TBARS levels were decreased compared with the LPS group.

Studies have reported that ATP release from immune cells occurs in subcellular areas where locally released ATP stimulates purinergic receptors that regulate functional cell responses (Ledderose et al. 2020).

It is defined that phosphocreatine systems are activated in specific organs and tissues that require high energy, such as skeletal muscle and the brain (Adriano et al. 2022). However, recent studies have shown that creatine is necessary to increase the activity of the immune system. One study showed that creatine supplementation supports the activity of CD8 + T cells in anti-tumor immunity by increasing ATP production (Di Biase et al. 2019). Also, recent studies suggest that ATP is an important signaling molecule for the activation of the immune system and that it may achieve this effect by stimulating P2X7 and suppressing purinergic receptor. While it is suggested that P2X4, from the purinergic receptor family, plays a role in immunomodulation, it is reported that P2X7 may trigger the inflammatory pathway (Sathanoori et al. 2015). In a study reported that extracellular ATP (released by endothelial and immune cells) and its metabolite ADP were shown to be important pro-inflammatory mediators through the activation of purinergic P2 receptors (P2Y and P2X), representing potential new targets for anti-inflammatory therapy. Also, it reported that melatonin is a negative modulator of endothelial purinergic P2Y1R signaling by inhibiting P2Y1R-mediated leukocyte adhesion and TNF-α production and exerting an anti-inflammatory effect (Cardoso et al. 2017). However, innate immunity has been reported to provide a potent front-line defense against various pathogenic invasions. Neutrophils are important innate immune cells that play a role in this defense mechanism. It has been reported that during the acute phase of inflammation, neutrophils mobilize to the infected site and aggressively eliminate bacteria through phagocytosis and by producing antibacterial agents (Cao et al. 2021). They quickly need sufficient energy resources to realize these abilities. In this context, ATP is the primary energy source and is known to be provided primarily through glycolysis rather than oxidative phosphorylation in mitochondria. It has been suggested that higher than normal neutrophil activity may be promoted if rapid ATP production under inflammatory conditions involves another metabolic reaction. A recent study suggested that creatine supplementation increases the immunological activity of neutrophils, and this is achieved by increasing the level of cellular ATP. This functional modification of neutrophils has been implicated in a mouse model of bacterial sepsis, contributing to reduced mortality due to increased antibacterial immunity (Saito et al. 2022). Since it occurs as a result of mitochondrial oxygen consumption, the conversion rate of ADP to ATP in the electron transport chain can be considered an indicator of the mitochondrial respiration rate. The organism uses creatine group molecules to maintain energy production with degraded ATP. Impairment of cellular respiration can lead to circulatory and perfusion disorders, secondary hypoxia, and multiorgan failure.

In parallel with the literature information, in our study, we found that there was a significantly decrease in ATP, creatine p levels in the LPS group compared with the control, melatonin, and LPS + Mel groups. ATP levels decreased in the LPS + Mel group compared with the control group. On the contrary, there was a significant increase in AMP, ADP, creatine levels, and ADP/ATP ratio in the LPS group compared with the control, melatonin, and LPS + Mel groups. However, in the LPS + Mel group, creatine levels increased significantly in the control and melatonin groups. These results show that melatonin improves the energy balance in adenosine and creatine metabolism, which is disrupted in the brain tissue due to sepsis. However, we also come to the conclusion that the effect of melatonin on adenosine metabolism is more effective than on creatine metabolism. The dose and application time of melatonin may have affected this effect.

Studies have reported that melatonin strengthens energy sources or provides alternative energy sources that may provide neurological protection against neurodegeneration caused by brain damage (Ikram et al. 2021). Various studies have shown that enhancing energy sensors through their kinases can provide neuroprotection after neurodegeneration. In a study, traumatic brain injury (TBI) decreased the expression of phospho-5′AMP-activated protein kinase (p-AMPK) and phospho-cAMP-response element binding (p-CREB) and activated the phosphorylation of NF-κB in brain tissue of mice with TBI (Hill et al. 2016). It has been reported that melatonin maintains energy levels and regulates mitochondrial functions by increasing p-AMPK and p-CREB expression and decreasing p- factor nuclear factor kappa beta (NF-κB) (Rehman et al. 2019).

Increased activity of the pro-inflammatory pathway following LPS administration is one of the main stimulants of oxidative stress and tissue damage. It is known that as a result of stimulation of the pro-inflammatory transcription gene factor NF-κß with LPS, many pro-inflammatory cytokines such as TNF-α are released and tissue damage and subsequent organ failure develop. Studies have shown that melatonin is effective on neuroinflammation and oxidative stress by reducing NF-Kß levels (Ikram et al. 2021). It is suggested that increased inflammatory activity in the brain tissue leads to the disappearance of the blood–brain barrier, especially in brain parts such as the hippocampus, and the resulting degeneration of neurons (Suwannakot et al. 2022). Neuron-specific enolase (NSE), which is used as a neuronal damage marker, has been suggested as an early stage marker in many diseases characterized by neuronal damage (Yao et al. 2014; Bangshøj et al. 2022). In the literature, NSE plays an important role in cell growth and survival and has been reported to exhibit both neurotrophic activity and neuroprotective effects. Therefore, NSE serves as a marker of neuronal damage in patients with traumatic brain injury and acute ischemic stroke. One study shows that the level of c-enolase was significantly reduced in vehicle-treated animals, while its expression in melatonin-treated animals was similar to that in sham-operated animals. Supporting these findings, another study showed that melatonin affects the proliferation and differentiation of neural stem cells (Moriya et al. 2007; Sung et al. 2009). In our study, we found that the NSE levels examined in serum were significantly higher in the group in which we induced sepsis with LPS compared with the other groups, and there was no significant difference between the other groups. In the immunofluorescence examinations, we carried out at the tissue level that we observed that s100-ß immunoreactivity, which marks the damaged neurons, increased significantly in the LPS group compared with the other groups, especially in the sections taken from the hippocampus, which is one of the regions where the blood–brain barrier is disrupted and one of the first areas affected by inflammation. On the contrary, we investigated that NeUn immunoreactivity, which we mark the number of living neurons, decreased significantly in the LPS group compared with the other groups. In the LPS + Mel group, the number of damaged neurons was found to decrease compared with the LPS group, while the number of live neurons was found to increase compared with the LPS group. In addition, in our study where we examined synaptophysin immunoreactivity to examine synaptic transmission, it was found that LPS application showed a decrease in synaptophysin immunoreactivity compared with the other groups, and melatonin application improved the synaptic transmission impaired in sepsis with the increase in synaptophysin retention. Studies have observed that melatonin application suppresses the inflammatory pathway, reduces caspase-3 activity, and increases the inflammation-related decrease in synaptophysin levels, one of the markers of presynaptic transmission in neuronal degeneration (Mustafa et al. 2020).

In our study, neuronal damage and loss of neurons resulting from sepsis-related neuroinflammation and oxidative stress in rats with sepsis induced by LPS were shown by markings in the tissue and markers in the serum. It was observed that melatonin application in the sepsis group reduced neuronal damage and loss and reduced oxidative stress and neuronal damage markers. We also observed that mitochondrial damage and energy balance disorders induced by oxidative stress in sepsis were improved by melatonin application and that melatonin had an energy-protective effect, as stated in the literature. We think that melatonin protects against sepsis-related neuroinflammation, neuronal damage, and energy balance disorders.

## Limitation

In our study where we observed the effects of melatonin on neuronal damage and energy balance in rats with sepsis, more advanced and specific molecular studies are needed to fully elucidate the mechanism.

## Data Availability

No datasets were generated or analysed during the current study.
