# Correction: Utility of melatonin on brain injury, synaptic transmission, and energy metabolism in rats with sepsis

**DOI:** 10.1007/s00210-025-04784-7

**Published:** 2025-11-20

**Authors:** Gulten Ates, Sule Tamer, Elif Ozkok, Hatice Yorulmaz, Gul Ipek Gundogan, Abdullah Aksu, Nuray Balkis

**Affiliations:** 1 Department of Physiology, Faculty of Medicine, Istanbul Yeni Yuzyil University, Yilanlı Ayazma St, Cevizlibag, Istanbul, 34010 Turkey; 2 Department of Physiology, Istanbul Medical Faculty, Istanbul University, Istanbul, Turkey; 3 Department of Neuroscience, Aziz Sancar Institute of Experimental Medicine, Istanbul University, Istanbul, Turkey; 4 Faculty of Health Science, Halic University, Istanbul, Turkey; 5 Department of Histology and Embryology, Faculty of Medicine, Biruni University, Istanbul, Turkey; 6 Department of Chemical Oceanography, Institute of Marine Sciences and Management, Istanbul University, Istanbul, Turkey

**Correction: Naunyn-Schmiedeberg's Archives of Pharmacology (2024) 398:1509-1519**

10.1007/s00210-024-03337-8

The published original article contained an error.

Local Ethics Committee (2017/63) should be corrected as Local Ethics Committee (2016/62)

The original article has been corrected.

